# How do providers of artificial intelligence (AI) solutions propose and legitimize the values of their solutions for supporting diagnostic radiology workflow? A technography study in 2021

**DOI:** 10.1007/s00330-022-09090-x

**Published:** 2022-08-18

**Authors:** Mohammad H. Rezazade Mehrizi, Simon H. Gerritsen, Wouter M. de Klerk, Chantal Houtschild, Silke M. H. Dinnessen, Luna Zhao, Rik van Sommeren, Abby Zerfu

**Affiliations:** 1 KIN Center for Digital Innovation, School of Business and Economics, Vrije Universiteit Amsterdam, De Boelelaan 1105, VU Main Building A-Wing, 5th Floor, 1081 HV Amsterdam, The Netherlands; 2 Digital Business and Innovation, Vrije Universiteit Amsterdam, Amsterdam, The Netherlands

**Keywords:** Artificial intelligence, Radiology, Value proposition, Legitimization

## Abstract

**Objectives:**

How do providers of artificial intelligence (AI) solutions propose and legitimize the values of their solutions for supporting diagnostic radiology workflow?

**Methods:**

We systematically analyze 393 AI applications developed for supporting diagnostic radiology workflow. We collected qualitative and quantitative data by analyzing around 1250 pages of documents retrieved from companies’ websites and legal documents. Five investigators read and interpreted collected data, extracted the features and functionalities of the AI applications, and finally entered them into an excel file for identifying the patterns.

**Results:**

Over the last 2 years, we see an increase in the number of AI applications (43%) and number of companies offering them (34%), as well as their average age (45%). Companies claim various value propositions related to increasing the “efficiency” of radiology work (18%)—e.g., via reducing the time and cost of performing tasks and reducing the work pressure—and “quality” of offering medical services (31%)—e.g., via enhancing the quality of clinical decisions and enhancing the quality of patient care, or both of them (28%). To legitimize and support their value propositions, the companies use multiple strategies simultaneously, particularly by seeking legal approvals (72%), promoting their partnership with medical and academic institutions (75%), highlighting the expertise of their teams (56%), and showcasing examples of implementing their solutions in practice (53%).

**Conclusions:**

Although providers of AI applications claim a wide range of value propositions, they often provide limited evidence to show how their solutions deliver such systematic values in clinical practice.

**Key Points:**

*• AI applications in radiology continue to grow in number and diversity.*

*• Companies offering AI applications claim various value propositions and use multiple ways to legitimize these propositions.*

*• Systematic scientific evidence showing the actual effectiveness of AI applications in clinical context is limited.*

**Supplementary Information:**

The online version contains supplementary material available at 10.1007/s00330-022-09090-x.

## Introduction

The development of artificial intelligence (AI) applications in the radiology domain has witnessed a high pace in the last few years [1]. Many pilot algorithms have been developed into commercial solutions and increasingly introduced into medical institutions. Nevertheless, still companies developing these algorithms face the challenge of defining a medically relevant and economically viable value proposition and convincing medical institutions and practitioners to adopt these solutions in clinical practice [2]. This has been a hurdle for adopting these solutions and developing among users [2].

From the perspective of technology-acceptance theory [3], both “proposing” medically relevant and economically viable values and “legitimizing” such value claims are essential for convincing users to adopt a technology. This is a challenge for many new technologies, especially for the case of AI solutions offered to the radiology domain. Proposing a medically relevant value is challenging since many AI solutions are narrow in terms of the functionality and tasks that they support, are still in their early stage of being developed and seamlessly integrated into the radiology workflow, and require heavy preparation work for being validated and adjusted to the users [1]. At the same time, justifying the value and ensuring the quality of AI solutions are difficult because the users are medical experts who have high level of quality expectation and jurisdictional concerns [4], especially due to the legal and ethical sensitivity of using AI applications [5].

Given that users’ trust in AI applications is one of the key drivers of adopting them [2, 5], it is important to analyze how these companies try to assure users about the credibility of their applications. These questions motivated our systematic technography study to examine how the providers of AI solutions in the radiology domain formulate the values of their AI solutions and in which ways they try to legitimize such value claims.

Several reports, opinion articles, and commercial reviews have tried to map out the AI applications [6, 7]. For instance, scholars have investigated the strengths of scientific evidence that developers of AI offer for supporting their solutions [7]. Although insightful, these studies often have a selective sample size, especially from commercially visible applications [7, 8] and thus miss out the wider range of AI applications that are part of the upcoming waves of technological solutions. In addition, these studies often have a narrow focus in terms of the value propositions and ways of legitimizing them.

To tackle these limitations, we conducted a systematic analysis of AI applications in the domain of radiology. As we explain in our methodology section, by using both qualitative and quantitative data from various sources, we captured the characteristics of these applications and mapped them particularly around four themes: (1) technological features, (2) developing companies, (3) value propositions of AI applications, and (4) ways of legitimizing AI value propositions.

In the next section, we describe our findings and discuss the implications of our findings for evaluating, implementing, and using AI applications in radiology work.

## Methods

We collected data by systematically reviewing companies and their AI applications in the domain of radiology. We started by the list of applications presented during the RSNA, ECR, and SIIM conferences, taking place 2017–2021. In addition, we searched in industrial and market research sources (e.g., Blackford Analysis), social media channels (LinkedIn and Twitter), and company websites (when referring to partners) to find other companies active in this domain. An application was deemed suitable for analysis if its goal was to support activities for any step in the diagnostic radiology workflow *and* if its functions were supported by machine learning algorithms. Examples of excluded applications were AI marketplaces for applications, solutions that only connected an image storage system (PACS) with an information system (RIS), or products which only processed non-medical data. We also excluded applications that were removed from the market or merged to form bundled offerings. Eventually this process resulted in 393 different applications offered by 133 companies (see Appendixes 1 and 2 for the list of included applications and companies).

For each application, we collected information regarding (1) the developing company and its team^1^, (2) their technological characteristics, (3) their clinical features and functionalities, (4) the various ways that companies prompted the values of their applications, and (5) the ways of ensuring the legitimacy of their applications. The data was gathered from various sources such as company websites, news articles, white papers, FDA approval documents, user instruction documents, and scientific publications. We also cross-checked the data and reviewed the credibility of the sources. Overall, the collected information consists of more than 10,000 pages. The data is up to date as of August 2021.

To analyze the data, we created qualitative case reports of each company and the applications it offered. We analyzed these documents using systematic thematic analysis [9] to code applications and companies based on a detailed codebook developed according to the four dimensions (see Appendix 3).

First, we offer a global overview of the AI *applications* and zoom into their technological features in terms of (1) types of algorithms and data sources used for developing these applications, (2) technological architectures they have (e.g., on-premise vs. cloud-based), and (3) how they are integrated into the workflow of radiologists (e.g., as embedded into the PACS systems). We further analyze the functionalities that these applications offer to their users, namely, for which imaging modalities these applications are useful, which anatomical regions are specialized for, what specific tasks and functions do they perform, and which steps of the radiological workflow do they support?

Second, we examine the *companies* active in developing and offering AI applications in radiology regarding their (1) demographic profiles such as their country of origin, age, and size, (2) core businesses (e.g., medical or technological), and (3) the founding teams and their expertise.

Third, we examine the range of value propositions that these companies claim in relation to their AI solutions, including the “efficiency”-related values such as reducing cost, time, and workload in the radiological workflow, and “quality”-related values such as enhancing the quality of clinical decisions and offering a higher quality of medical services.

Fourth, we analyze how the companies legitimize the value propositions, through seeking external credibility (e.g., having legal approvals and formal partnerships with medical and academic institutions; promoting the financial, technical, and human resources and expertise they have; referring to scientific evidence related to their AI solutions; and showcasing the implementations of their systems in practice).

Following the coding procedure described in [9] and to ensure the accuracy and consistency, the coding was executed by five post-graduate researchers, trained in digital innovation and particularly AI solutions in healthcare. Each researcher coded 20% of the variables for all the cases and cross-checked 5% of the data coded by another coder. The results were checked and integrated by a professor of digital healthcare, who discussed the potential inconsistencies and resolved them among the coders. All coding variables were equally divided among the coders, and each coder was assigned a peer. At the end, a senior researcher checked the coded data and recoded 20% of the data to ensure the quality and consistency of the coding, thus ensuring above 80% of inter-rater reliability and resolving the disagreements. Throughout the coding, seven peer meetings were scheduled to discuss the coding.

## Results

In this section, we describe the results of our technography study conducted in August 2021 and we compare these results with the similar situation in August 2019 [1] to show the changes and trends.

### 1) Overview of AI applications developed in the domain of radiology

**Fig. 1:**
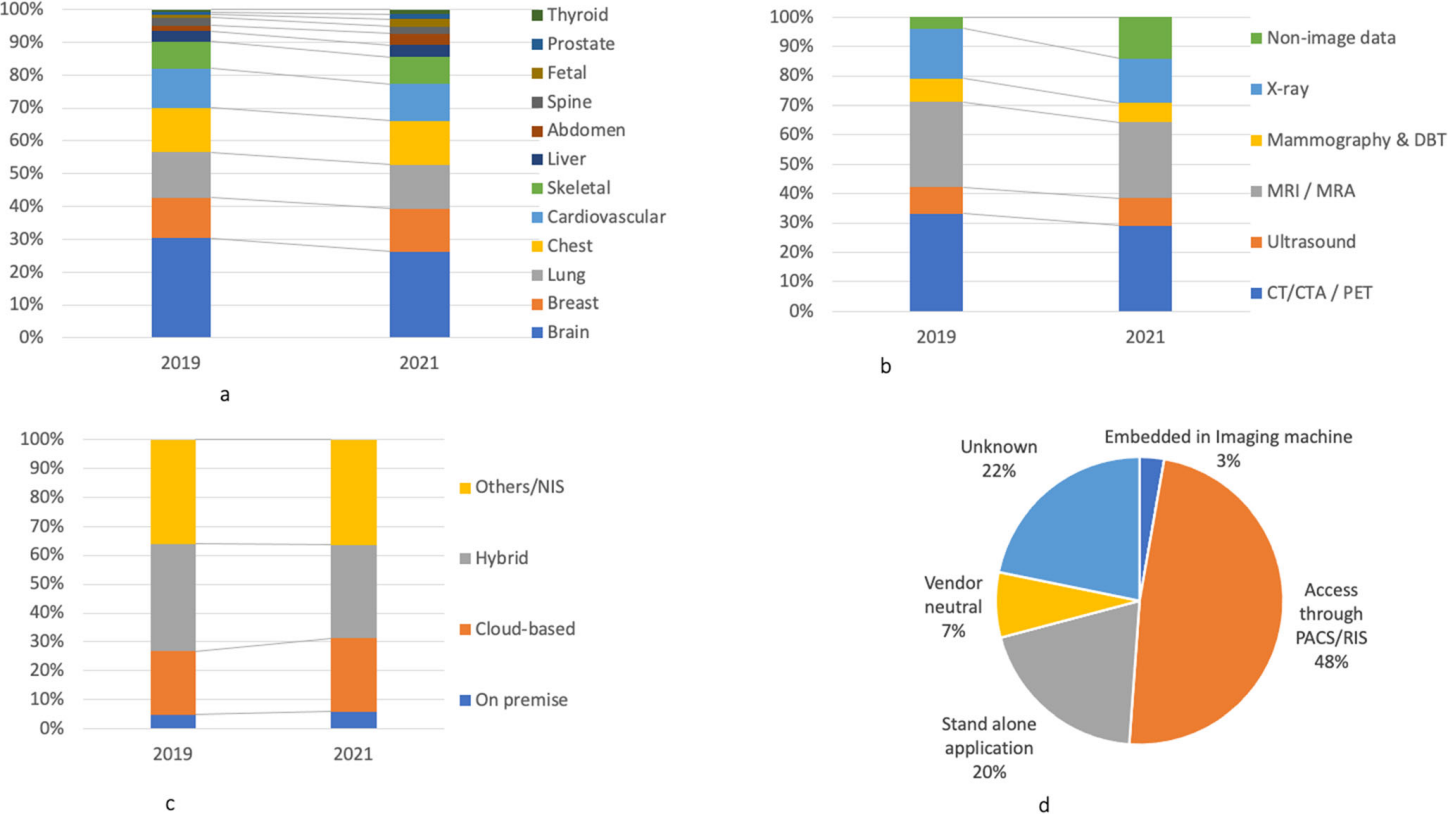
Characteristics of AI applications in radiology domain

We identified 393 AI applications offered by 133 companies, showing a growth of 43% and 34% respectively since August 2019. From this number, 227 (58%) are commercially available in the market, 103 (26%) are pending for receiving legal approvals, and 64 (16%) are still under development (see Fig. 1).

#### Technological characteristics of AI applications

**Fig. 2:**
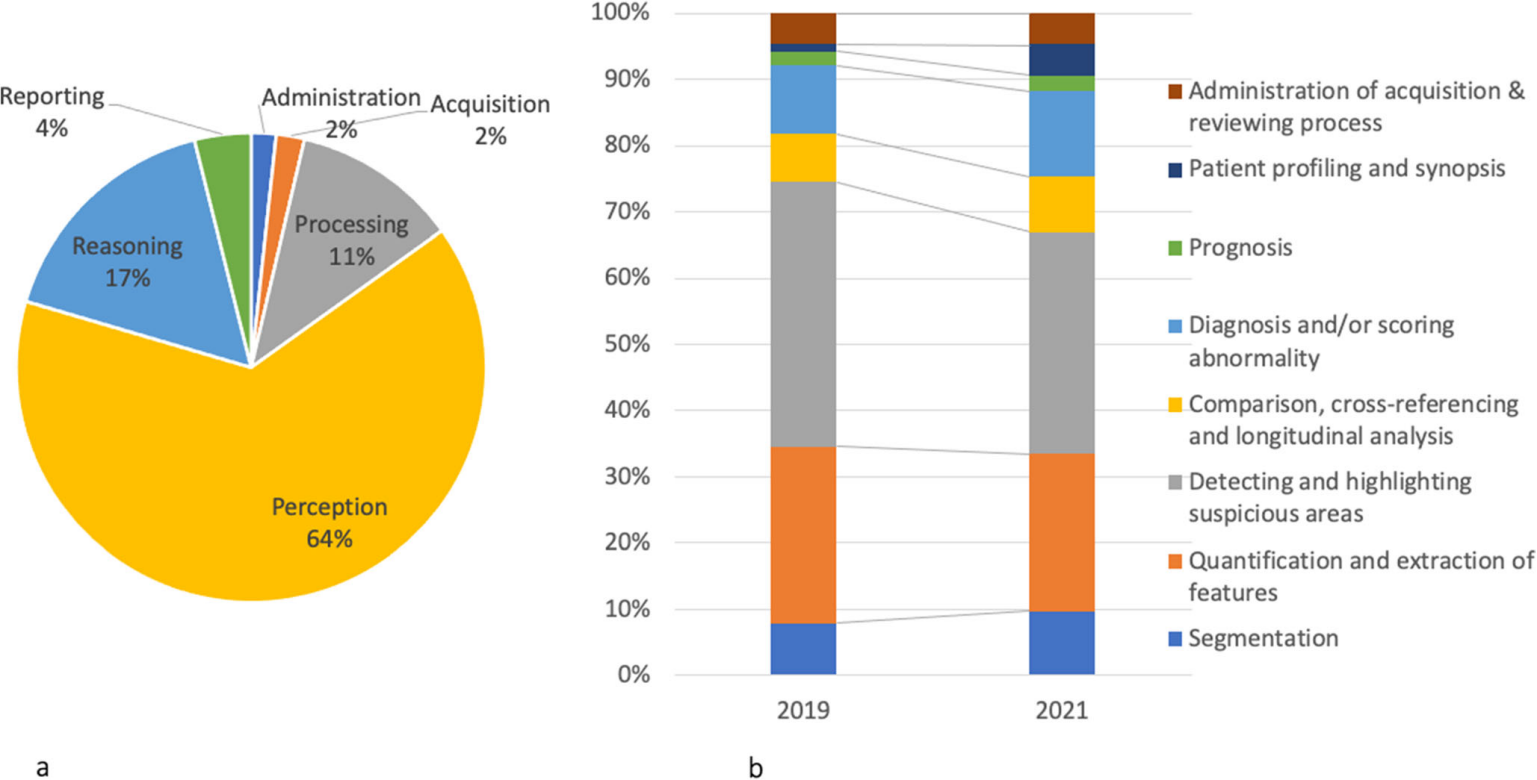
The contribution of AI applications to radiology workflow

Usually, each application works with one specific image modality, on which the algorithm is trained. The most common imaging modalities are CT scan and MRI. The number of AI applications using non-imaging data (e.g., using clinical information and genomic data) has increased since 2019 (Fig. 2).

Although all the applications use a form of machine learning algorithms, only half of them explicitly indicate using “deep learning” algorithms, whereas others generally refer to “machine learning” (14%) or do not indicate the algorithm type (35%). Only 20 applications (6%) specify the type of deep learning algorithm they use. The data sources used for training algorithms are disclosed for only 8% of the applications and only 10% indicate the quantity of their training dataset, with a range from 1000 to more than 1,000,000 images (Appendix 4).

Regarding their architecture, 48% are integrated into the radiological working environment (e.g., PACS/RIS), 20% are accessed as stand-alone applications, 7% as vendor-neutral applications are available on various working environments, and 3% are embedded into the scanning machines (20% of the applications do not specify this matter). As for their technical configuration, 25% are cloud-based, 32% are hybrid (both cloud-based and on-premise), and 6% are only on-premise. The trend shows that AI applications prioritize cloud-based architecture over being hybrid or purely on-premise.

#### Clinical features and functionalities of AI applications

AI applications are specialized in terms of the clinical features and functionalities that they support. Although the existing applications cover a wide range of anatomical regions, each application focuses on one specific region, such as “brain” (21%), “chest area in general” (11%), “lung” (11%), “breast” (10%), and “cardiovascular regions” (9%). Compared with 2019, “brain” is less dominant as the focal region, and other regions such as “abdomen,” “prostate,” and “thyroid” are more popular.

When examining their functionalities, the applications primarily focus on “detecting and highlighting suspicious areas” (33%) and “quantifying and extracting features” (24%). There is an increase in the share of applications developed for “diagnosis and scoring abnormalities” (13%), “segmentation” (10%), and “patient profiling and synopsis” (5%) over the last 2 years. Regarding the steps of radiological workflow, we see that 64% of the applications support “perception” tasks, followed by “reasoning” (17%), and “image processing” (11%). The share of applications supporting “reporting” task has increased since 2019 (from 2 to 4%). As for the legal approval, 50% of the applications have at least one approval, mainly CE-marked (38%) and FDA-cleared (32%). Compared with 2019, more applications have both FDA and CE approvals.

### 2) Overview of the companies active in offering AI applications in radiology

**Fig. 3:**
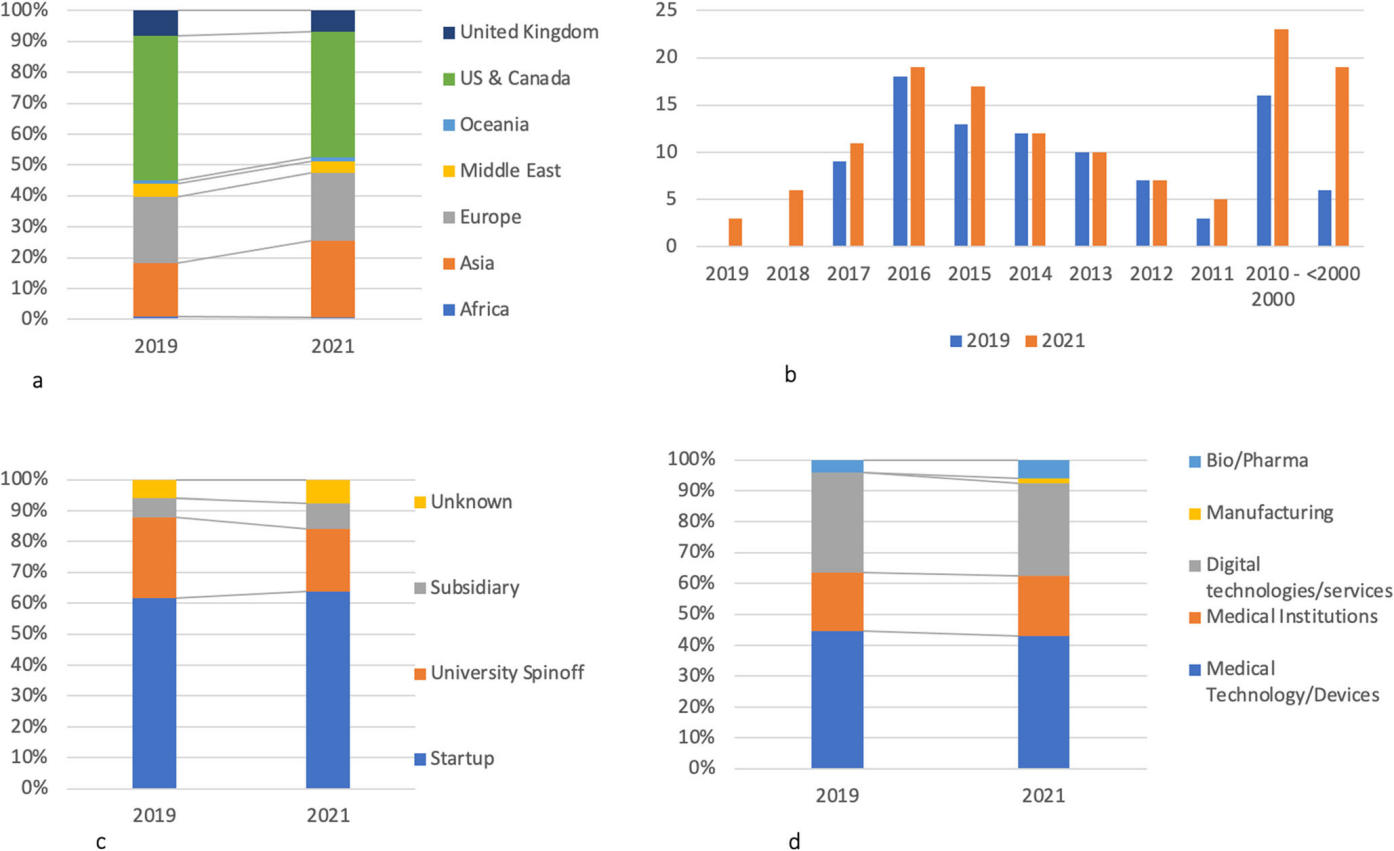
Characteristics of companies active in developing AI applications

The companies active in offering AI applications have their headquarters mainly in North America (41%), Asia (25%), and Europe (22%). The share of Asian companies has increased, from 17% in 2019 to 25% in 2021. Their sizes range from micro (18%), small (44%), medium (21%), to large (17%). Since 2019, the percentage of “large” firms has increased from 10 to 20%. Their ages range between 2 and 154 years, with an average of 15 years. More than 70% of the companies are younger than 11 years old. The percentage of the grown-ups (5–9 years old) has increased from 36% in 2019 to 52% in 2021. Many startups managed to survive in the market for longer than 5 years. The average number of applications per company is 3, which has increased since 2019 (2, 7). The number of applications offered by companies ranges from 1 to 38, and 70% of the companies offer up to 6 applications (see Fig. 3).

As for their core business, medical technology/device companies are leading in offering AI applications (43%), followed by digital technology/service companies (30%) and medical institutions (20%). Originally, 64% of companies are startups and 20% are spinoffs from university and research institutions. Forty-three percent of the founders have technical background (such as software development and engineering), 25% have a medical background, and 12% have a hybrid background in both medical and technical domains (e.g., doctors with training in computer science).

### 3) Various value propositions of AI solutions in the radiology domain

**Fig. 4:**
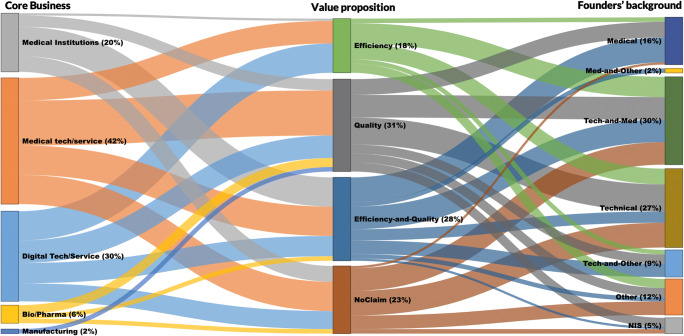
Value propositions claimed by companies related to AI applications

When these companies promote their AI applications, they claim different, and often multiple, “value propositions,” related to increasing the “efficiency” of radiology work (18%)—e.g., via reducing the time and cost of performing tasks and reducing the work pressure—and enhancing the “quality” of offering medical services (31%)—e.g., via enhancing the quality of clinical decisions and improving the quality of patient care, or both of them (28%). Twenty-three percent of the companies do not explicitly claim any of these value propositions (see Table 1 and Fig. 4).

**Table 1:** Value propositions claimed by companies offering AI applications

Value proposition	Related themes	Example quote
Making better decisions (33%)	Evidence-based decision support, enhance clinical decision-making	“HealthMyne’s proprietary algorithms automate the extraction of quantitative imaging metrics at the Point-of-Read, greatly reducing inter- and intra-reader variability and making evidence-based metrics including volume, density, mass, doubling-times, heterogeneity, and others available at the Point-of-Care.” (HealthMyne)
		“Our products were designed to help medical imaging experts make confident decisions with clearer information and convenient decision guidance.” (ClariPI, Inc.)
Offering higher quality of care for patients (31%)	Improved patient outcomes, saving lives, improve patient lives	“Bridge human and artificial intelligence to advance medical diagnostics to improve patient outcomes around the world. Facilitate a world in which radiologists are empowered with the most advanced medical diagnostics tools to facilitate optimal patient care and support.” (Enlitic)
		“This enables physicians to see the information hiding in the images and drive data-based, personalized patient care decisions from diagnosis, to therapy tracking, to planning for procedures.” (Imbio)
Speeding up the work (19%)	Saving time, improve speed of diagnostic, faster decision-making	“QIR (CE marked) is a cardiac MRI analysis software which allows the doctor’s working time to be reduced by a factor of 10 and which covers all his clinical needs while including a unique solution.” (CASIS)
		“Blackford’s products work seamlessly within existing systems to enable instant comparison of multiple image studies with a single click, providing a typical time-saving of 10%-20% for each comparison made.” (BlackFord Analysis)
Reducing the cost of work (10%)	Decreasing costs, reduction of revenue loss, less expensive healthcare	“The company’s software is used for faster diagnosing of X-Ray, MRI and CT images using computer-aided diagnostics tools that makes diagnoses cheaper.” (Aidence)
		“Our Investment for better and less expensive healthcare: Detecting and measuring clinical conditions in musculoskeletal MRI.” (Balzano Informatik AG)
Reducing work pressure for medical practitioners (7%)	Addresses shortage of radiologists, avoid burnouts, clients can focus on what they do best	“We want to lift some weight off the physician’s shoulders, doing the time consuming, uninspiring data analyzing for them. Think of white matter hyperintensities getting segmented for you, but not without you checking whether the computer was right and having the opportunity of correcting.” (Quantib BV.)
		“Our solutions are designed to augment human expertise and improve clinical and operational workflows, allowing our clients to focus on what they do best.” (IBM Watson Health)

Companies with founders with technical background (35) rarely claim both quality and efficiency values at the same time (5), and it is likely that they do not make any explicitly claim (11). Companies who have their founders (partly) with the medical background (62) tend to claim more quality-related values (17) than efficiency-related values (11). Companies making both efficiency and quality claims in 95% have radiologists involved in their core team.

### 4) Ways of legitimizing the values of AI applications

**Fig. 5:**
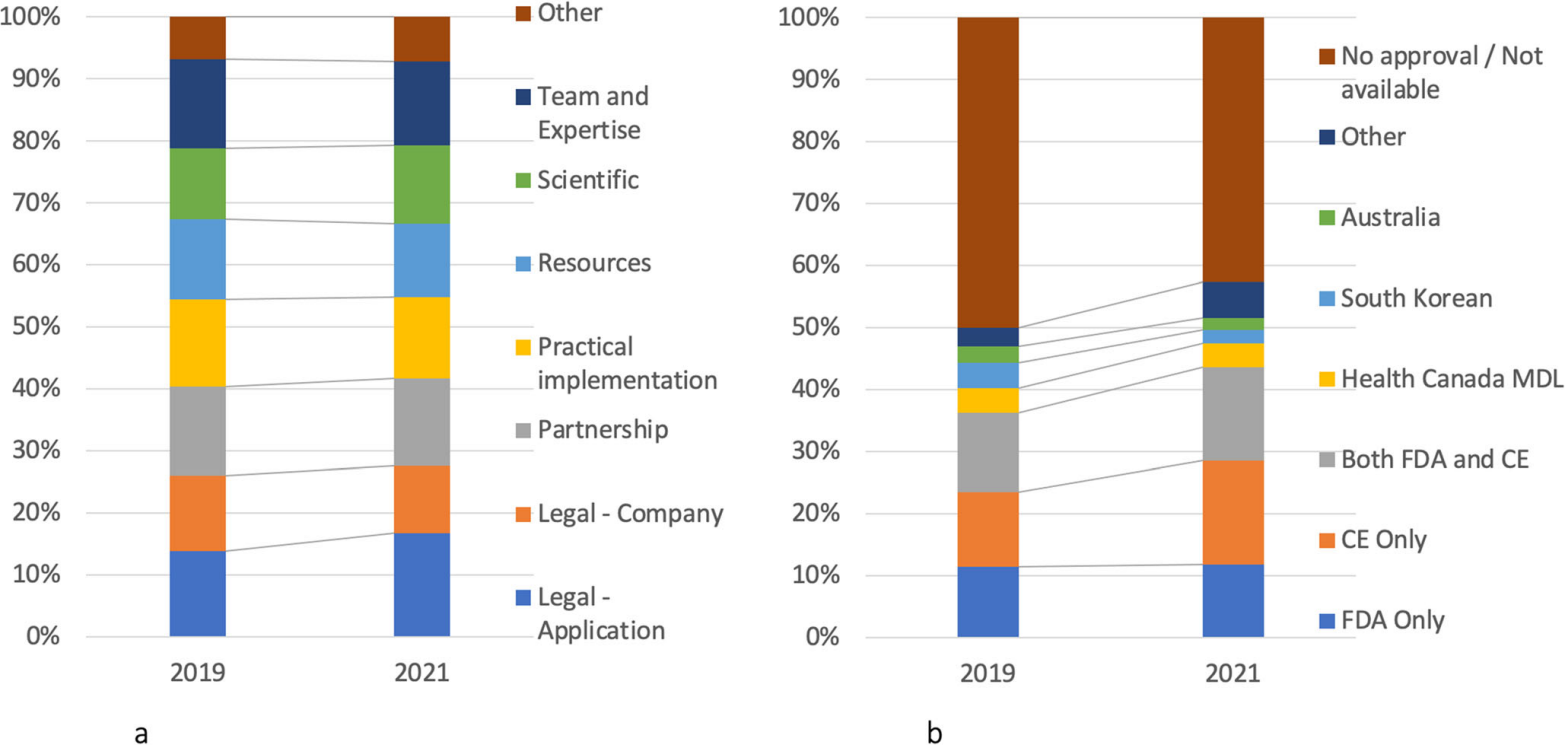
Ways of legitimizing AI applications and their legal approvals

**Fig. 6:**
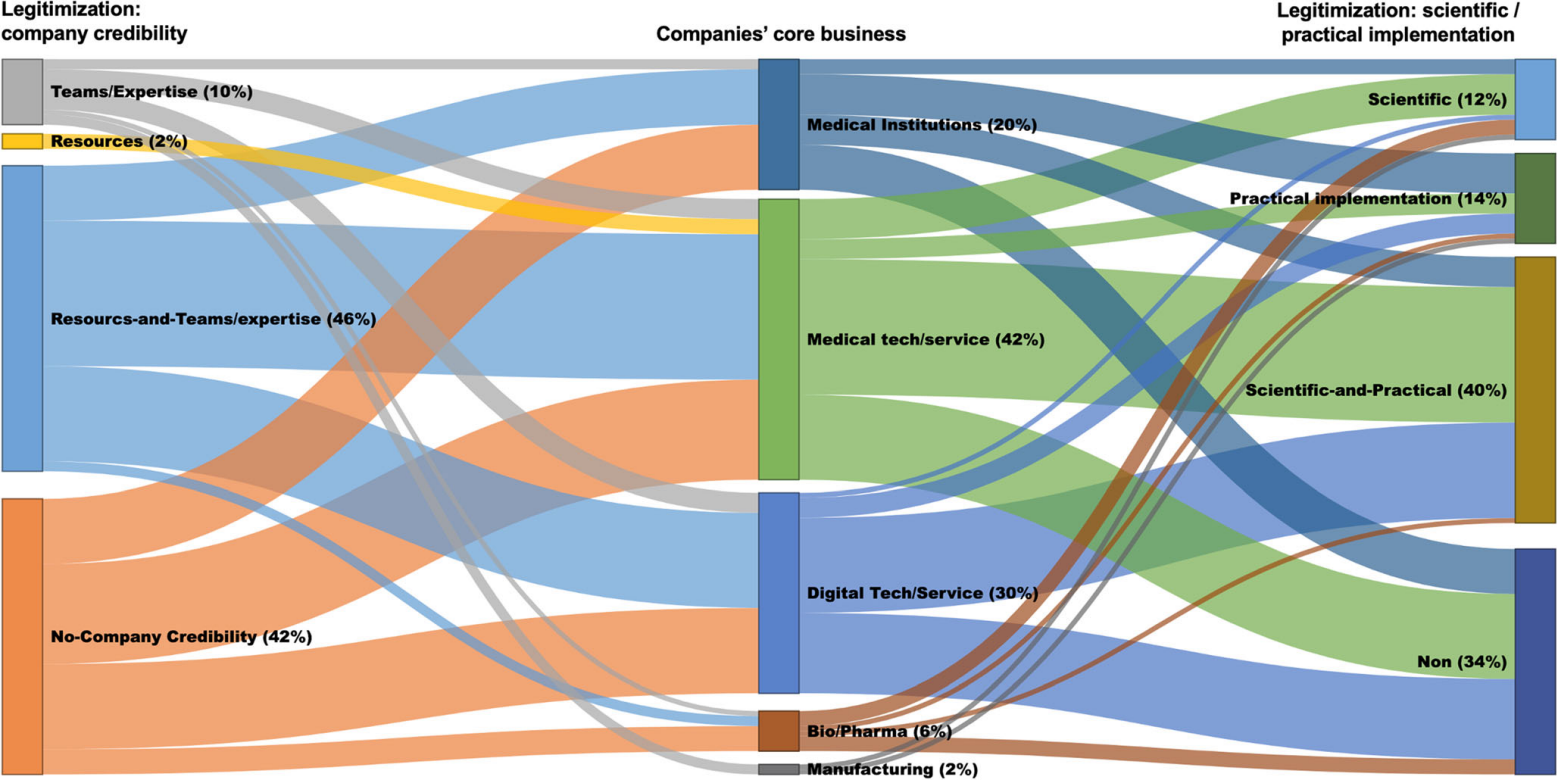
Different legitimization strategies used by companies providing AI applications

The companies use various ways to legitimize and support the value propositions of their AI applications (see Table 2 and Figs. 5 and 6), namely, by (1) seeking external legitimacy—i.e., obtaining legal approvals (72%) and forming partnerships with medical and academic institutions (75%), (2) promoting the financial and technological resources of their company (48%) as well as the human expertise in their teams (56%), (3) showing scientific evidence and their engagement in scientific research in relation to their solutions (52%), and (4) showcasing examples of implementing their solutions in practice (53%). Often companies use several of these legitimization strategies simultaneously.

**Table 2:** Ways of legitimizing AI applications and their companies

Theme	Description	Example quote
External credibility - legal	Referring to formal approvals or certifications for their applications or for the entire company	“Volpara Health Technologies Limited holds certification to ISO27001 and ISO13485; MDSAP 690110 certification for Japan, Australia, Canada, and the United States; and is a US FDA–registered establishment whose products are listed accordingly” (Volpara Solutions)
		“This system is the first FDA-cleared 3D tomosynthesis software using AI to aid in breast cancer detection.” (iCAD)
External credibility - partnership	Referring to partnerships as a way to legitimize their products	“Siemens Healthineers, which has partnerships with all 15 of the best US hospitals, offers a powerful research infrastructure encompassing a growing data pool of more than 100 million curated medical images and four data centers with one petaflops of computing power.” (Siemens Healthineers)
		“The partnership provides access to UPH’s extensive and growing multi-state network of hospitals and clinics, and to its network of clinical experts who will contribute valuable insights to VIDA’s solution roadmap and commercialization processes. The partnership uniquely scales VIDA to expand and strengthen its portfolio of clinically impactful solutions.” (VIDA)
Company credibility: resources	Referring to the financial and technical resources to signal competency of the company	“The Aidoc solution uses Amazon Elastic Compute Cloud (Amazon EC2) P3 instances to train machine learning (ML) models and execute inference processes; Amazon Simple Storage Service (Amazon S3) to store anonymized medical imagery for analysis; and Amazon Relational Database Service (Amazon RDS) to store image metadata.” (Aidoc Medical Ltd.)
		“Almost every week investors knock on the doors in Nijmegen. Van Rikxoort and Van Ginneken (co-founders) choose to use their own capital to grow at their own pace.” (Thirona) (Translation)
Company credibility: resources	Promoting the expertise and affiliations of their team members	“With the QView team’s experience in artificial intelligence, they developed sophisticated algorithms and underwent rigorous testing with a comprehensive training set to achieve acceptable performance in diagnostic accuracy.” (QView)
		“With a strong and experienced team of scientists, ScreenPoint developed the AI system Transpara, which matches the performance of experienced radiologists in detecting breast cancer in screening mammograms.” (ScreenPoint Medical)
Scientific	Highlighting research papers and results to support applications	“The icobrain AI solutions are already used in more than 100 hospitals and imaging center networks worldwide, and in clinical studies by 4 out of the Top 5 pharmaceutical companies.” (Icometrix)
		“DBT is an evolutional in digital mammography systems, with initial clinical evidence indicating a higher cancer detection rate, particularly in women with dense breasts, and a lower false-positive recall rate.” (Konica Minolta)
Practical implementation	Showcasing the range and type of implementations of the applications	“By being vendor agnostic, customers don’t have to settle for one vendor’s software. That means you can choose what you want – as well as access it from anywhere.” (NeoSoft LLC)
		“The integration of cNeuro® cMRI into Siemens Healthineers Digital Ecosystem allows its users to seamlessly upload images to cNeuro® cMRI, view results and save a report.” (Combinostics)
Other	Other ways of showcasing the legitimacy such as commitment to customer needs	Customer commitment: “Thousands of institutions across 74 countries trust us to deliver the best solutions for advanced visualization and artificial intelligence." (TeraRecon)
		Claims of being a world leader: “most advanced and only clinically proven software platform for cerebrovascular disorders - worldwide leader in advanced imaging for stroke - Secure and Accessible” (RapidAI)

Seeking external legitimacy seems to be default legitimization strategy since 87% of the companies who make a value claim offer a form of external credibility. Companies are more distinguishable based on whether they promote their scientific engagement or practical implementations, or both. Medical institutions tend to make efficiency-and-quality claim less frequently than the other companies, and scientific legalization seems to be more favored among pharmaceutical/bio companies, compared with other companies. The older the companies, the more likely they are to use both scientific and practical implementation to legitimize their value propositions.

## Discussions

By combining qualitative and quantitative data from various sources, we mapped out the diversity of the AI applications, the companies active in developing them, and the ways in which these applications are claimed to contribute to radiology work and how the companies try to legitimize their offerings.

Our analysis shows that the companies offering AI applications have become more mature as the shares of large firms and grown-up companies have increased. In addition, the skewedness towards focusing on certain anatomical regions (e.g., brain) has reduced over the last 2 years and companies have expand their range of algorithms working with different forms of medical data. In addition, there is a more balanced distribution of companies across North American, European, and Asian regions. These all indicate that more substantial resources and efforts have been invested in developing AI applications, and the market is beyond its initial emerging phase.

Nevertheless, the AI applications are still highly specialized and narrow in terms of imaging modality, the anatomical regions, and medical functionalities. It seems that the main strategy of companies for dealing with the narrowness of their applications is to diversify their applications (offering more solutions) and ensure their solutions are seamlessly integrated into the working environment of radiologists and being operable on multiple technological infrastructures.

The companies rarely (less than 10%) disclose the technical information about the type of algorithm they use and the training datasets. This might be because many of the companies are still in the process of improving their algorithms and enhancing the richness of the data on which their algorithms can perform well. More transparency on these aspects is crucial for creating trust and critical engagement of the users who want to know how these algorithms are trained and how they can be used on their own data [10].

Companies claim various values that their applications offer for supporting radiology workflow, both for increasing the efficiency and quality of clinical work. This shows that companies have learned how to define value propositions which are practically relevant for medical professionals and managers [11] to be able to justify the implementation of these applications from financial and safety standpoints [12].

To support these value propositions, companies use a wide range of legitimization strategies. Having some form of external legitimacy (e.g., legal approval or partnerships with medical and academic institutions) seems a rather prerequisite condition, though often complemented with promoting the company credibility (e.g., based on their resources and expertise) as well as showcasing their engagement in scientific research and the implementation of their solutions in practice. Nevertheless, companies often stay at the level of showing the accuracy and performance of their algorithms on the training and testing data, without offering systematic evidence on the effectiveness of their solutions in the clinical practice.

## Conclusion

We provided a systematic overview of how the market of AI applications in radiology has evolved. We see that the market has been expanded in terms of the number and diversity of the AI applications and companies are more active in defining various forms of value propositions and legitimizing their claims. However, systematic evidence showing the performance and effectiveness of these solutions on the users’ side, such as clear evidence on the reduction of the time or costs or increasing the quality, is limited. This is a crucial step to ensure that AI applications offer clinically relevant and practically viable value [13]. It is important to also recognize that there are many AI initiatives by both industrial R&D teams and academic labs, which we did not cover in our study. Hence, future research needs to extend our study to include the upcoming AI solutions and companies as well as examining how the existing ones evolve.

## Supplementary information

ESM 1 (XLSX 655 kb)

ESM 2 (XLSX 210 kb)

ESM 3 (DOCX 43 kb)

## Abbreviations

AI: Artificial intelligence
CE mark: European conformity marking
CT: Computed tomography
CTA: Computed tomography angiography
ECR: European Conference of Radiology
EU: European Union
FDA: Food and Drug Administration
MRA: Magnetic resonance angiography
MRI: Magnetic resonance imaging
NA: North America
PACS: Picture Archiving and Communication System
PET: Positron emission tomography
RIS: Radiological information system
RSNA: Radiological Society of North America
SIIM: Society for Imaging Informatics in Medicine
US: United States
X-ray: X-radiation

## Footnotes

1 During the coding process, we categorized company size based on the taxonomy presented by the Organization for Economic Cooperation and Development (OECD). This classification is as follows: “Micro”: fewer than ten employees, “Small”: 10 to 49 employees, “Medium”: 50 to 249 employees, and “Large”: more than 250 employees.

## Declarations

### Guarantor

The scientific guarantor of this publication is Prof. Marleen Huysman (m.h.huysman@vu.nl).

### Statistics and biometry

No complex statistical methods were necessary for this paper.

### Methodology

• multicenter study (as a review of all applications available in the market)
