# Supplementary material for: How do providers of artificial intelligence (AI) solutions propose and legitimize the values of their solutions for supporting diagnostic radiology workflow? A technography study in 2021
Source: Eur Radiol. 2022 Aug 18;33(2):915–24. doi: 10.1007/s00330-022-09090-x (PMC9889424; doi:10.1007/s00330-022-09090-x)
Supplement: Supplementary file 3 — (DOCX 43 kb) [file 330_2022_9090_MOESM3_ESM.docx]

Table A1. Codebook used for coding the data related to AI applications and the companies

| **Company level** | **Description** |
| --- | --- |
| Company name |  |
| Number of applications | The number of AI applications that the company offers on its website |
| Foundation year |  |
| Headquarter location | The country/region where the company’s headquarter locates |
|  |  |
| Size | Company sizes are classified by the number of full-time employees: Micro (<10), Small (10-49), Medium (50-249) Large (250+) |
| Core business | Computer software, Medical devices, Hospital & Health care, Information Technologies, AI & Internet, Biotechnology, Electrical & Electronic manufacturing, Mechanical & Industrial engineering, Pharmaceuticals |
| Origin | How the company was founded in the first place, e.g., as a spin off of the university, as a startup, as a spin off of a large medical company, … |
| Expertise of the funders: what is the expertise of the company founders | |
| Technical | Founder had most experience in for example: Data scientists, computer scientists, Artificial Intelligence experts, Robotic experts, etc. |
| Medical | Founder had most experience in for example: Radiologist, Neurologist, Cardiologist, Surgeon, etc. |
| Technical & Medical | Founder had experience in Biomedical engineering or an equal amount of experience in both areas |
| Use of domain experts (radiologists) in the team | Whether the company involves radiologists in its activities such as R&D, Testing, Validation, Design & Implementation, Customer Management. |
| Value proposition: Proposing how they contribute to radiology work through their AI applications | |
| Efficiency- Speeding up | Claiming reduced time spent on a specific task (e.g., segmentation or measurement) |
| Efficiency: cost reduction | Claiming reduced cost of performing tasks (e.g., by requiring fewer hours of having human actors performing it) |
| Efficiency: work pressure | Claiming reduced work pressure on medical practitioners (e.g., reduced workload of the radiologists for reporting normal cases) |
| Quality: making better decisions | Claiming that using AI applications help medical practitioners making more accurate decisions (e.g., reducing the error and increasing the accuracy of staging tumors). |
| Quality: increased quality of patient care | Claiming that using AI applications lead to higher quality of medical services for the patients (e.g., engagement of the patient in the medical diagnosis, more information for the patients, reducing the wait-time for patients) |
| **Ways of legitimizing the AI applications** | |
| External credibility- Legal | Referring to formal approvals or certifications for their applications or for the entire company |
| External Credibility- Partnership | Referring to partnerships with medical or academic institutions |
| Company credibility: Resources | Referring to the financial and technical resources to signal competency of the company |
| Company credibility: Team / Expertise | Promoting the expertise and affiliations of their team-members |
| Scientific | Highlighting research papers and results to support applications |
| Practical implementation | Showcasing the range and type of implementations of the applications |
| Other | Other ways of show-casing the legitimacy such as commitment to customers needs |

| **Application level** | **Description** |
| --- | --- |
| Technology-AI | Machine or Deep learning used for the algorithms, sometimes companies were more specific in their algorithms like indicating CCN, ANN or rule-based techniques. |
| Data | The amount of data was used for training and the sources from which the data was gathered |
| Targeted step in the workflow: Supporting which tasks in the radiological workflow | |
| Admiration | A step in the workflow concerning administrative tasks regarding content delivery, invoice processing, departmental data sharing, etc. |
| Acquisition | The creation of a representation of the visual characteristics of an object, also called digital imaging. |
| Processing | Enhancing image quality by performing operations (e.g. adjusting contrasts pr optical density) on the acquired image. |
| Perception | Allocating characteristics, features and metrics to the image in order to provide insights into the meaning of the image leading to a diagnosis. |
| Reasoning | The diagnosis and the underlying reason indicating why this decision is being made. |
| Reporting | The output structure providing insights in the results of the diagnosis. |
| Functionalities | What specific tasks and actions does the AI application enable us to perform: Segmentation, Quantification and extraction of features, Detecting and highlighting suspicious areas, Comparison, cross-referencing and longitudinal analysis, Diagnosis and/or scoring abnormality, Prognosis, Patient profiling and synopsis, Administration of acquisition & reviewing process |
| Anatomical region | Which anatomical region does the AI application is specialized on: Brain, Breast, Lung, Cardiovascular, Liver, Spine, (Musculo) Skeletal, Thyroid, Prostate, Chest, Abdomen, Fetal |
| State of development | Is the application still under development, under approval, or already approved |
| Architecture | Is the application running on premise, cloud-based, both or in a hybrid format. |
| Integration in workflow | Is the application embedded into a certain imaging machine, is it accessible through PACS or RIS, is it stand-alone, is it vendor neutral |
| Legal approvals | Indicate whether this specific application has received clearance for different regions and by different regulatory institutions. |

Table A2. The common data sources used for training AI applications

| **Data source used for training AI applications** | **Frequency** |
| --- | --- |
| Unknown medical center | 32 |
| Unknown private (own) database | 11 |
| Unknown database | 7 |
| DBT manufacturers | 6 |
| Partners | 6 |
| Community of Clinicians | 5 |
| OEM (machines) vendor | 4 |
| Other company systems | 3 |
| EHR database | 2 |
| Stanford University | 2 |
| Japanese Society of Radiological Technology and the U.S &    National Library of Medicine | 1 |
| Messidor database | 1 |
| U.K.’s National Health Service | 1 |
